# Diagnostic Performance of Line-Field Confocal Optical Coherence Tomography for Basal Cell Carcinoma: A Prospective Study

**DOI:** 10.3390/cancers18010153

**Published:** 2026-01-01

**Authors:** Carmen Orte Cano, Dilara Sanak, Clément Lenoir, Gwendoline Diet, Margot Fontaine, Lucas Boussingault, Lyna Mtimet, Dina Aktas, Stefan Rusu, Anne-Laure Trepant, Gerardo Palmisano, Alessandro Di Stefani, Elisa Cinotti, Linda Tognetti, Javiera Pérez-Anker, Ketty Peris, Pietro Rubegni, Susana Puig, Josep Malvehy, Jean-Luc Perrot, Véronique Del Marmol, Mariano Suppa

**Affiliations:** 1 Department of Dermatology, Hôpital Erasme, Hôpital Universitaire de Bruxelles (HUB), Université Libre de Bruxelles, 1070 Brussels, Belgium; 2 Department of Dermato-Oncology, Institut Jules Bordet, Hôpital Universitaire de Bruxelles (HUB), Université Libre de Bruxelles, 1070 Brussels, Belgium; 3 Department of Pathology, Hôpital Erasme, Hôpital Universitaire de Bruxelles (HUB), Université Libre de Bruxelles, 1070 Brussels, Belgium; 4 Department of Pathology, Centre Universitaire inter Régional d’Expertise en Anatomie Pathologique Hospitalière (CurePath), 6000 Charleroi, Belgium; 5 Dermatology Unit, Dipartimento Universitario di Medicina e Chirurgia Traslazionale, Università Cattolica del Sacro Cuore, 00168 Rome, Italy; 6 Dermatology Unit, Dipartimento Scienze Mediche e Chirurgiche, Fondazione Policlinico Universitario A. Gemelli IRCCS, 00168 Rome, Italy; 7 Dermatology Unit, Department of Medical, Surgical and Neurological Sciences, University of Siena, 53100 Siena, Italy; 8 Groupe d’Imagerie Cutanée Non Invasive (GICNI), Société Française de Dermatologie (SFD), 75006 Paris, France; 9 Melanoma Unit, Hospital Clinic Barcelona, University of Barcelona, 08036 Barcelona, Spain; 10 CIBER de Enfermedades Raras, Instituto de Salud Carlos III, 08036 Barcelona, Spain; 11 Department of Dermatology, University Hospital of Saint-Etienne, 42055 Saint-Etienne, France

**Keywords:** line-field confocal optical coherence tomography (LC-OCT), basal cell carcinoma (BCC), diagnostic performance

## Abstract

**Background**: Line-field confocal optical coherence tomography (LC-OCT) is an innovative non-invasive imaging technique recently introduced in dermatology. Its use has been established particularly in the diagnosis of skin cancer. The LC-OCT description of basal cell carcinoma (BCC) criteria has served for the conduction of retrospective evaluations on its diagnostic performance, which demonstrated its utility in diagnosis and subtyping BCC. However, prospective studies performed at patients’ bedside are currently lacking in this field. **Objective**: The objective of the study was to provide parameters of LC-OCT diagnostic performance for BCC derived from a prospective study performed at patients’ bedside. **Methods**: Lesions clinically equivocal for BCC were prospectively included. Dermoscopic and LC-OCT diagnoses were obtained at the patients’ bedside by an expert observer prior to surgical excision or biopsy. **Results**: A total of 214 lesions (163 BCCs and 51 BCC imitators) belonging to 119 patients were included. For the differentiation of BCC from BCC-imitators, LC-OCT had the same sensitivity as dermoscopy (98%) but a better specificity (90% vs. 37%). For the discrimination of superficial BCC from other BCC subtypes, LC-OCT had an increased sensitivity and specificity compared to dermoscopy (72% vs. 62%, and 97% vs. 84%, respectively). **Conclusions**: This prospective study showed that the diagnostic performance for BCC diagnosis and subtyping can be remarkedly increased by LC-OCT compared to dermoscopic examination alone. Our data encourages the inclusion of LC-OCT in the diagnostic process and management of equivocal lesions for BCC.

## Simple Summary

Basal cell carcinoma (BCC) is the most common form of skin cancer worldwide. Although it is rarely life-threatening, it often develops on the face, which can cause significant aesthetic concerns for patients. Its high prevalence is also associated with substantial healthcare costs. Diagnosing and subtyping BCC is key to treatment choice—topical therapies can be used for superficial BCC, while deeper forms usually require surgery—but this process typically relies on invasive biopsy. Line-field confocal optical coherence tomography (LC-OCT) is a novel non-invasive “virtual biopsy” that supports diagnosis and subtyping. In this prospective, real-life study of equivocal lesions, LC-OCT improved diagnostic confidence and showed greater accuracy than dermoscopy. The main diagnostic pitfall was confusion with sebaceous glands, while subtyping challenges included superficial trabeculated BCCs and BCC nodules extending beyond the LC-OCT’s field of view. These findings support LC-OCT as a bedside alternative to biopsies to guide treatment.

## 1. Introduction

Basal cell carcinoma (BCC) is classified together with squamous cell carcinoma as a non-melanoma skin cancer and is a complex disease in which ultraviolet radiation and individual factors (such as skin phototype, age, and immune status) interact [1]. It is the most common malignancy in dermatology with an increasing incidence [2,3]. Despite its low mortality rates, it may account for a significant morbidity due to invasion of the surrounding tissues with consequent ulceration and bleeding in delayed or high-risk cases or to consequences related to treatment in highly visible areas such as the head, face, and neck. It is a frequent issue of the dermatological practice that presents a significant economic burden for health care systems [4].

BCC can present as different morphological subtypes: superficial (sBCC), nodular (nBCC), and aggressive forms, including micronodular, morphoeic, and infiltrative (iBCC) [1]. Diagnosing and subtyping BCC can sometimes be challenging on clinical and dermoscopic examination alone [5,6]. These lesions can be described as “equivocal” when “a diagnosis of skin cancer is not possible to render or to exclude clinically and/or dermoscopically with absolute confidence” [7]. The differential diagnosis is wide, including benign lesions.

Various treatment options are available and recommended depending on the BCC subtype. In the case of clinical suspicion, current guidelines advise performing a punch biopsy to confirm the diagnosis and then choosing treatment depending on subtype and patient preferences [1]. Although surgery remains the cornerstone of treatment, in some cases (sBCC) patients could benefit from nonsurgical alternatives such as cryotherapy, topical therapies, or photodynamic therapy [1]. Correctly diagnosing and subtyping BCC is therefore important to ensure the best treatment, as this will have prognostic and aesthetic consequences. However, guidelines also stated that non-invasive imaging [including reflectance confocal microscopy (RCM) and/or optical coherence tomography (OCT)] should be used, when available, to improve the diagnostic accuracy in equivocal lesions [1]. The advantage of RCM is the high lateral resolution (1 µm), while the advantages of OCT are the high penetration (1–2 mm) and the vertical slice imaging. However, these techniques have some limitations; mainly, RCM has a limited penetration depth (250 µm) and provides images only on the horizontal plane (contrary to histopathology), whereas OCT features a low lateral resolution (7.5 µm) that prevents cellular visualisation.

Recently, line-field confocal optical coherence tomography (LC-OCT) was developed to meet the need for an in vivo non-invasive skin imaging device combining high resolution and deep penetration: LC-OCT produces live skin imaging with a ~1 µm isotropic resolution and up to a ~500 µm depth [8,9,10]. This tool seems to be, then, particularly suitable for non-invasive BCC diagnosis and subtyping. Indeed, shortly after its development, BCC was described under LC-OCT. First, the morphological characteristics of BCC and its subtypes in LC-OCT were studied by Suppa et al. [11]. Then, Ruini et al. observed similar features with an agreement of 90.4% between LC-OCT and histopathology for BCC subtyping, meaning LC-OCT and histopathology are comparable, and that the morphological characteristics described for BCC under LC-OCT correlate with those described in histopathology [12]. This was confirmed by Boussingault et al., who found an agreement between LC-OCT and histopathology ranging between 86.6% and 98.4%, with the most important criteria (BCC lobules and blood vessels) displaying 100% agreement rates [13]. Later, based on those reported morphological criteria, retrospective evaluations on LC-OCT diagnostic performances for BCC diagnosis [14] and subtyping were conducted [15,16,17].

To date, there are no reports on the accuracy of LC-OCT for BCC diagnosis and subtyping on prospectively evaluated data at patients’ bedside. The aim of the present prospective study was to determine the diagnostic accuracy of LC-OCT compared to the dermoscopic examination for the diagnosis and subtyping of BCC in a real-life setting.

## 2. Materials and Methods

This was a prospective observational study conducted at the Dermatology Department, Hôpital Erasme, Université Libre de Bruxelles, Brussels, Belgium, from 2020 to 2023. Subjects presenting with a lesion clinically equivocal for BCC, for which either a biopsy or excision was planned, were included. Exclusion criteria were recurrent or previously treated lesions. The study protocol was approved by the local ethical committee (P2020/393/B4062020000121) and was conducted in accordance with the Declaration of Helsinki and the International Conference on Harmonization Good Clinical Practice. All patients provided written informed consent.

Each study lesion was evaluated immediately after inclusion by a single observer who formulated both dermoscopic and LC-OCT diagnoses at patient’s bedside (therefore blinded to any histopathological data) according to previously established dermoscopic [5,6] and LC-OCT criteria for BCC [11,12], as it is performed in real-life practice, and not retrospectively (i.e., they could not be changed afterwards). A total of 3 observers (Observer-1: MS; Observer-2: CL; Observer-3: GD) with at least 3 years of experience in dermoscopy and LC-OCT participated in the evaluation of the study; however, each lesion was independently assessed by only one observer at the patient’s bedside, with no repeated evaluation by different investigators. LC-OCT images and videos were acquired with a CE-marked LC-OCT device (deepLive™, DAMAE Medical, Paris, France).

The investigators were asked to report their level of diagnostic confidence (0–100%) for each examination (dermoscopic and LC-OCT). A ≥90% threshold was chosen a priori to define ‘high diagnostic confidence’. After image acquisition, a biopsy or excision was performed in all cases, including those with no suspicion of BCC on LC-OCT. Histopathological diagnosis was used as the gold standard for diagnosis. For reasons of practicality, only the most aggressive subtype for each BCC was considered for this analysis, as it determines the patient management (i.e., in the case of superficial-nodular BCC, a nBCC was retained as the diagnosis; in the case of superficial-infiltrative BCC, nodular-infiltrative BCC, and superficial-nodular-infiltrative BCC, iBCC was retained as the diagnosis). Micronodular forms were considered iBCC.

To calculate the sample size, the following was considered: BCC prevalence 0.70; power 0.90; I-type error 0.05; dermoscopy accuracy 0.90 [18]; LC-OCT accuracy 0.96 (based on our experience, later validated by peer-reviewed data) [14,15,16,17]. The estimated sample size was 214 lesions (150 BCCs, 64 BCC imitators) according to Alonzo et al. [19].

Descriptive data and continuous variables are presented as medians with intervals. Specificity, sensitivity, positive predictive value (PPV), negative predictive value (NPV), and accuracy of LC-OCT for BCC diagnosis and subtyping were compared to the dermoscopic examination using MacNemar’s test (paired data). No adjustment on test multiplicity was performed, as only the comparison of specificities was expected to demonstrate differences. The analyses were carried out with SAS^®^ (version 9.4).

## 3. Results

### 3.1. Study Sample

In total, 214 lesions from 119 patients were included [60 (50.4%) females and 59 (49.6%) males; phototype I-IV (most commonly II and III, 75.6% and 16.8%, respectively)] with a median age of 66.4 (32.4–89.4) years. The median number of lesions per patient was 1 (1–18). The 214 included lesions that were either excised (95.3%) or biopsied (4.7%).

Of the 214 included lesions, 163 (76.2%) were BCCs, and 51 (23.8%) were BCC imitators. The 163 studied BCCs included 50 (30.6%) sBCCs, 80 (49%) nBCCs, and 33 (20.2%) iBCCs. The 51 BCC imitators included 7 (13.7%) sebaceous hyperplasias, 6 (11.8%) squamous cell carcinomas, 6 (11.8%) actinic keratoses, 6 (11.8%) dermal naevi, 3 (5.9%) seborrheic keratoses, 2 (3.9%) Bowen’s diseases, and 21 (41.2%) lesions classified as “other imitators” (including 3 cases of nonspecific dermal inflammation, and 1 case each of dermatofibroma, neurofibroma, trichoblastoma, nodular melanoma, verrucous papilloma, wart, lichen-planus like keratosis, tumour of the infundibulum, acrochordon, xanthogranuloma, spongiotic dermatitis, stasis dermatitis, acantholytic dyskeratosis, focal hyperothokeratosis, folliculitis, and scar).

### 3.2. Level of Diagnostic Confidence

#### 3.2.1. LC-OCT Increased the Level of Diagnostic Confidence

The mean diagnostic confidence for BCC diagnosis increased by 21.6% when using LC-OCT compared to dermoscopy (95.6% vs. 74.0%, respectively). The mean diagnostic confidence for BCC subtyping was increased by 18.0% when using LC-OCT compared to dermoscopy (93.9% vs. 75.9%, respectively).

#### 3.2.2. LC-OCT Increased the Number of Lesions Diagnosed with High Confidence

Of the 214 included lesions, 70 (32.7%) were diagnosed with high confidence when using dermoscopy, while 189 (88.3%) were diagnosed with high confidence when using LC-OCT (55.6% increase).

#### 3.2.3. LC-OCT Increased the Number of BCCs Subtyped with High Confidence

Dermoscopy allowed for a high confidence subtyping in 44/163 (27.0%) of the study BCCs, while LC-OCT examination allowed for a high confidence subtyping in 148/163 (90.8%) cases (63.8% increase).

### 3.3. Diagnostic Performance

#### 3.3.1. BCC Diagnosis: Differentiating BCC from Imitators

Data on the diagnostic performance for BCC differentiation from imitators is displayed in Table 1. Compared to dermoscopy (sensitivity 98%, specificity 37%, accuracy 83%), LC-OCT presented a higher diagnostic performance (sensitivity 98%, specificity 90%, accuracy 96%), meaning a 53% increase in specificity and a 13% increase in accuracy (*p* < 0.001). Sensitivity remained equal for both examinations. When considering only the 189 lesions diagnosed with high confidence, the diagnostic performance of LC-OCT for BCC diagnosis increased further (sensitivity 99%, specificity 94%, accuracy 98%). Dermoscopy yielded 83% PPV and 83% NPV, whereas LC-OCT achieved 97% PPV and 94% NPV.

#### 3.3.2. BCC Subtyping

Overall, the percentage of correctly diagnosed BCC subtypes was 58.9% by dermoscopy and 71.2% by LC-OCT. Details on the diagnostic performance for BCC subtype discrimination are displayed in Table 2. For the differentiation of sBCC from other subtypes, LC-OCT showed 72% sensitivity, 97% specificity and 89% accuracy. Compared to dermoscopy, all parameters increased significantly (*p* = 0.03): 10% increase in sensitivity, 13% increase in specificity and 12% increase in accuracy. A significant increase in diagnostic accuracy was also observed for the differentiation of iBCC from other subtypes (*p* = 0.001). For the differentiation of nBCC from other subtypes, a non-significant increase in diagnostic accuracy was observed (*p* = 0.45). Similar results were obtained when restricting the analysis to the 148 BCCs diagnosed with high confidence, both for their differentiation from clinical imitators and for their subtype discrimination. LC-OCT showed significantly higher PPV and NPV than dermoscopy for sBCC and iBCC. For nBCC, LC-OCT also showed higher PPV and NPV, although not significantly.

#### 3.3.3. Details on Diagnostic Errors

**Figure 1:**
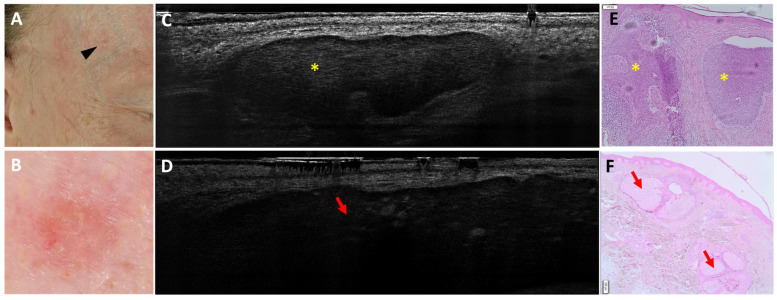
Coexistence of a nodular basal cell carcinoma and a sebaceous hyperplasia on the right temple of a 49-year-old man (black arrowhead, (**A**)): clinical (**A**), dermoscopic (**B**), LC-OCT vertical planes (**C**,**D**), and histopathological images (**E**,**F**). A small pink palpable lesion, characterised dermoscopically by a pink background with telangiectasias, showed different patterns on LC-OCT: a lobule separated from the epidermis featuring a triad of colours, corresponding to a nodular BCC (yellow star; panel (**C**)); and a bigger lobule separated from the epidermis characterised by granular-lobular pattern and loss of signal in the deepest area, corresponding to a sebaceous gland (red arrow; panel (**D**)). In histopathology, lobules of nodular BCC (yellow stars) are located next to sebaceous glands (red arrows). This case demonstrates the coexistence of two lobular structures within the same lesion.

**Figure 2:**
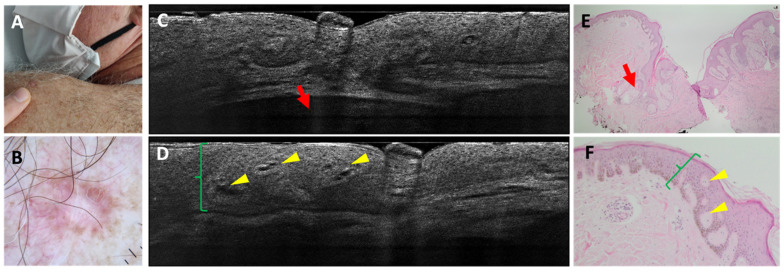
Dermatofibroma on the left shoulder of a 64-year-old man: clinical (**A**), dermoscopic (**B**), LC-OCT vertical images (**C**,**D**), and histopathological images (**E**,**F**). A pink firm papule over the left posterior shoulder was characterised dermoscopically by central scarring and peripheral pigment with scattered short-fine telangiectasias. LC-OCT examination showed acanthosis (green brackets) that can be mistaken for a superficial BCC because of the cleft-like appearance at the level of the dermal-epidermal junction. The deeper hypo-reflective lobule displays a deep loss of signal and corresponds to a sebaceous gland (red arrow). Histopathology shows a dermatofibroma characterised by an acanthosis (green brackets) forming intra-epidermal dermal islets (yellow arrows) and by the presence of a collagenous stroma. Next to the lesion are localised hair follicles with their respective sebaceous glands (red arrows).

Regarding the differentiation of BCC from imitators, 3 false negatives (FN) out of 163 BCCs were found; in all cases, the misclassification was due to the coexistence of an nBCC and a sebaceous hyperplasia within the same lesion (Figure 1). In total, 5 false positives (FP) out of 51 imitators corresponded histopathologically to 1 dermatofibroma (Figure 2), 1 scar, 1 actinic keratosis, and 2 nonspecific dermal inflammations.

**Figure 3:**
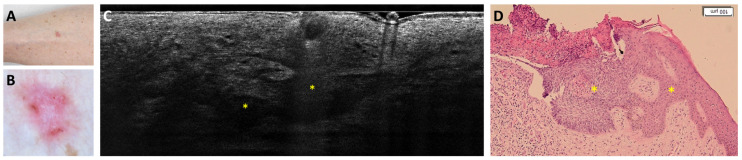
Trabecular superficial BCC on the anterior right leg of a 74-year-old woman that was misclassified as superficial and infiltrative BCC: clinical (**A**), dermoscopic (**B**), LC-OCT vertical image (**C**), and histopathological images (**D**). Clinical and dermoscopic pictures showed a pink macule with white streaks and short-fine telangiectasias. LC-OCT showed a superficially located branched lobule corresponding to a trabecular superficial BCC (yellow stars) in histopathology.

**Figure 4:**
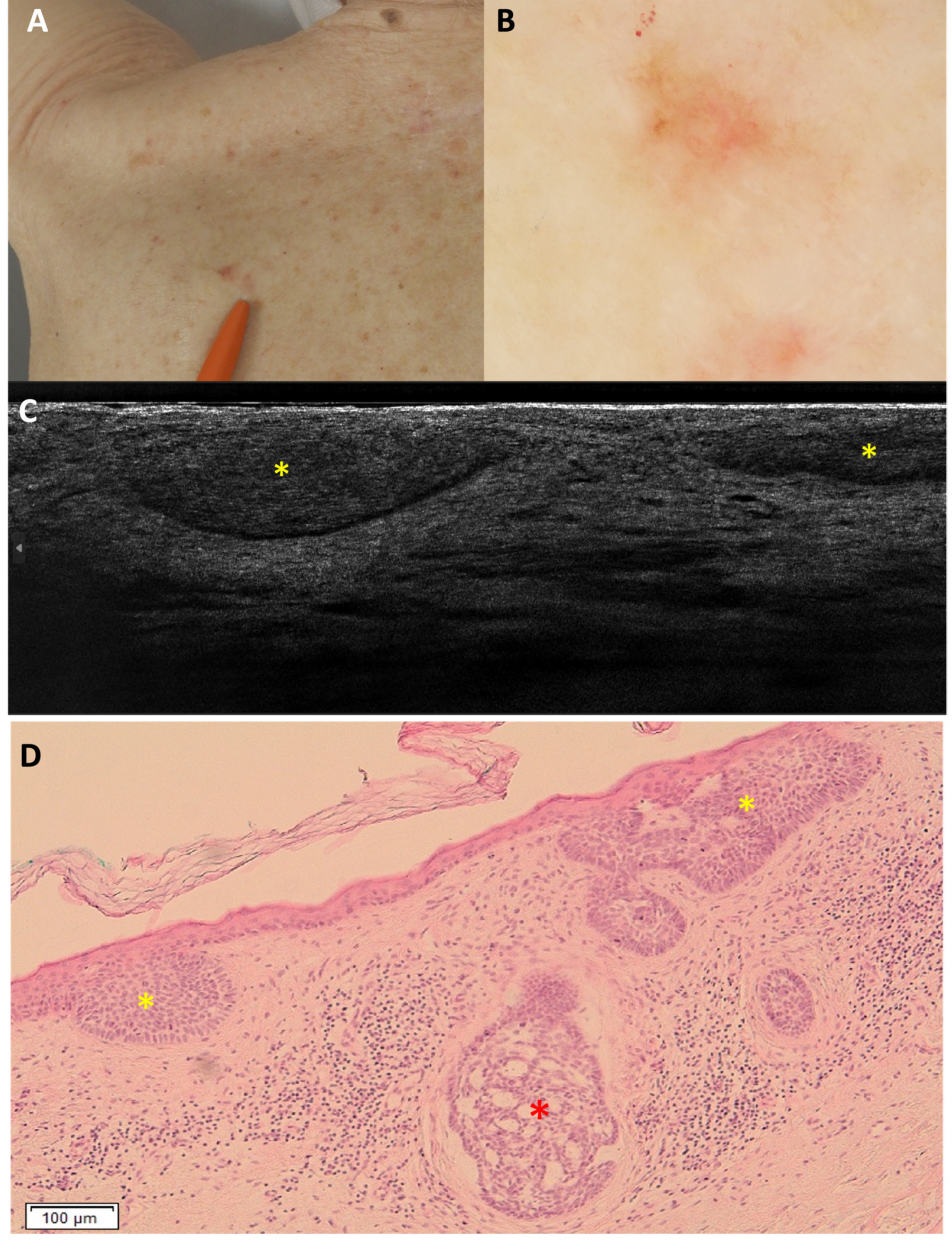
Superficial and nodular basal cell carcinoma on the right medial scapular region of an 80-year-old woman, which was underdiagnosed as only superficial: clinical (**A**), dermoscopic (**B**), LC-OCT vertical image (**C**), and histopathology (**D**). The lesion was dermoscopically characterised by a pigmented upper part, central white streaks and in-focus telangiectatic vessels in the lower part. LC-OCT showed hemispheric lobules connected to the epidermis, leading to the diagnosis of a superficial basal cell carcinoma. However, in histopathology, hemispheric lobules corresponding to a superficial component (yellow stars) as well as a deeper round lobule corresponding to a nodular component (red star) of the basal cell carcinoma were visualised.

**Figure 5:**
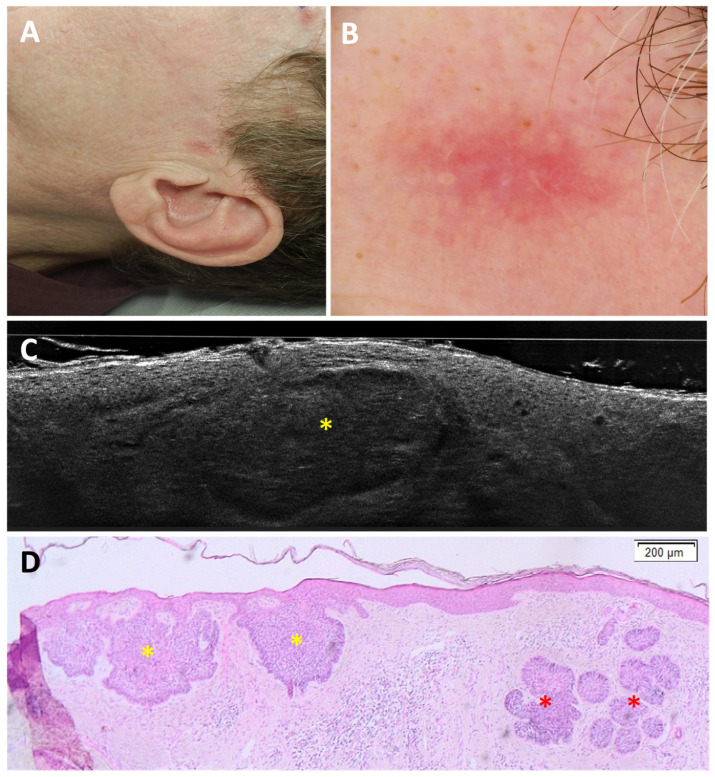
Superficial and nodular basal cell carcinoma on the left pre-auricular region of a 49-year-old man, which was underdiagnosed as superficial basal cell carcinoma: clinical (**A**), dermoscopic (**B**), LC-OCT vertical image (**C**), and histopathology (**D**). A pink macule was dermoscopically characterised by white streaks on a pinkish background. LC-OCT showed only hemispheric lobules (yellow star) connected to the epidermis. However, in histopathology, hemispheric lobules corresponding to a superficial component (yellow stars) as well as deeper round lobules corresponding to a nodular component (red stars) of the basal cell carcinoma were visualised.

Regarding sBCC subtyping, 14 FN were obtained: 11 trabecular sBCC were misclassified as iBCC (Figure 3), and 3 as nBCC; 4 FP were reported: 3 were classified as nBCC and 1 as iBCC in histopathology (Figure 4 and Figure 5).

**Figure 6:**
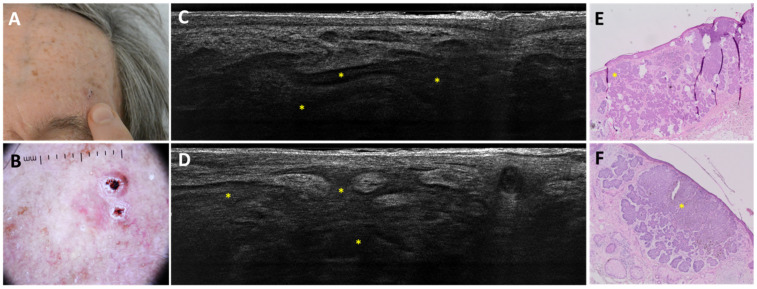
Coalescent nodular BCC on the left forehead of an 84-year-old man that was misclassified as infiltrative BCC: clinical (**A**), dermoscopic (**B**), LC-OCT vertical planes (**C**,**D**), and histopathological images (**E**,**F**). Clinical and dermoscopic images revealed a crusty maculo-papular lesion with a focalised peripheral pigmented area and arborising telangiectasias. LC-OCT showed branched coalescent lobules (yellow stars). Histopathology displayed a coalescent nodular BCC (yellow stars).

Regarding nBCC subtyping, 14 FN were obtained: 12 were misclassified as iBCC (Figure 6) and 2 as sBCC; 20 FP were reported: 17 were classified as iBCC and 3 as sBCC in histopathology.

**Figure 7:**
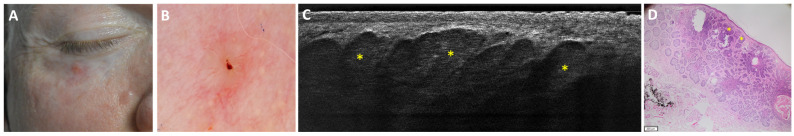
Micronodular basal cell carcinoma on the lower right eyelid of a 49-year-old man that was misdiagnosed as a nodular basal cell carcinoma with LC-OCT: clinical (**A**), dermoscopic (**B**), LC-OCT vertical plane (**C**), and histopathological image (**D**). A pink papule on the lower right eyelid was characterised by in-focus arborising vessels and a central excoriation on dermoscopy. LC-OCT displayed multiple adjacent round lobules (yellow stars) corresponding to a micronodular BCC in histopathology.

Regarding iBCC subtyping, 18 FN were obtained: 17 were misclassified as nBCC (Figure 7) and 1 as sBCC; 23 FP were reported: 12 were classified as nBCC and 11 as sBCC in histopathology.

### 3.4. Inter-Observer Variability

Of the 214 study lesions, 119 (55.6%) were evaluated by Observer-1, 54 (25.2%) by Observer-2, and 41 (19.2%) by Observer-3. Across the three observers, diagnostic confidence and diagnostic performance remained consistent, with no significant inter-observer variability observed in the outcomes. As each study lesion was independently assessed by only one observer with no repeated evaluation by different investigators, inter-observer agreement could not be assessed in this study.

## 4. Discussion

As recent retrospective evaluations suggested the usefulness of LC-OCT for the diagnosis and subtyping of BCC [14,15,16,17], we conducted the first real-life prospective observational study in which all diagnoses were made at the patient’s bedside by LC-OCT experts.

For the differentiation of BCC from imitators, the sensitivity of dermoscopy and LC-OCT were equal (98%) in our study. The high sensitivity obtained by dermoscopy proves the right selection of equivocal lesions and, for both tools, confirms their convenience as screening instruments. When comparing their specificity, an outstanding 53% increase with LC-OCT was achieved, confirming its usefulness in refining the diagnosis of equivocal lesions by reducing the FP rate. In line with our previous retrospective study [16], LC-OCT increased the diagnostic accuracy by 13% (15% when considering high-confidence lesions only) compared to dermoscopy. In our cohort, dermoscopy achieved a PPV and NPV of 83%, indicating that both positive and negative diagnoses were correct in more than four out of five cases. Conversely, LC-OCT reached 97% PPV and 94% NPV, suggesting that a positive LC-OCT diagnosis of BCC was almost always correct and that a negative result was highly reliable.

Until now, the literature included four studies reporting the diagnostic performances of LC-OCT for BCC based on retrospective databases [14,15,16,17]. Gust et al. collected lesions prospectively but analysed images retrospectively [15]. Cinotti et al. [17] and Donelli et al. [14] reported real-life performances on a retrospectively analysed database. Mtimet et al. produced a diagnostic algorithm for BCC diagnosis and subtyping in the largest retrospective study [16]. In each of these past studies, LC-OCT demonstrated high sensitivity (97–98%) [14,15,16,17]. In line with our data, the study by Cinotti et al. found no significant changes between the sensitivities of dermoscopy and LC-OCT for BCC diagnosis [17].

The high specificity (>90%) of LC-OCT for BCC diagnosis observed in this study is consistent with previous reports, except for Gust et al. [15], who found an 8% lower specificity. In contrast, other studies reported increases ranging from 9% [17] up to 53% (as in the present study). The lower specificity found by Gust et al. could be explained by the fact that the number of lesions for which the operator reported a high diagnostic confidence was lower than in other studies (70% vs. 94.1% in Mtimet et al., and 88.3% in this study) [15,16]. Indeed, when considering only high-confidence lesions, Gust et al. reached a specificity of 97% [15]. Moreover, their analysis was conducted at a time when several studies on LC-OCT criteria for imitators were not yet available [20,21,22].

The diagnostic accuracy of LC-OCT for BCC was similar across studies (>90%) [14,15,16,17]. This means an increase over dermoscopy from 3% [15] to the 13% reached in this study.

Concerning diagnostic performances of other non-invasive imaging techniques, a meta-analysis including 15 studies on the diagnostic performances of RCM for BCC demonstrated a sensitivity of 92% (73–100%) and specificity of 93% (38–100%) [23]. In a later study, RCM reached a sensitivity and specificity of 99% and 59.1%, respectively [24]. The significant variability in the sensitivity and specificity results of RCM attests to the lack of reproducibility among centres, depending on their level of expertise. Available data on the use of LC-OCT for the diagnosis of BCC appears uniform across studies, suggesting a good reproducibility of the technique. Indeed, LC-OCT is easy to use and interpret, providing intuitive histology-like vertical images and RCM-like horizontal images with cellular-level resolution [25].

Diagnostic performances of conventional OCT in the field of BCC have been described in a meta-analysis including three studies, reporting a sensitivity of 95% (91–97%) and a specificity of 77% (69–83%) [26]. Another meta-analysis including 31 studies showed an overall 89.3% sensitivity and 60.3% specificity [27]. A more recent study reported 95.7% sensitivity and 73.1% specificity [28]. These results appear to be less variable than those obtained with RCM, proving the reproducibility of the results in the field of OCT. However, when comparing LC-OCT to conventional OCT, a much higher specificity is obtained with LC-OCT, pointing to its superiority in terms of diagnostic accuracy.

Discriminating between sBCC and other subtypes is of clinical relevance as sBCCs are candidates for non-surgical treatments. In this context, LC-OCT showed a significant 12% increase in diagnostic accuracy compared to dermoscopy. Previously reported diagnostic performances of LC-OCT for sBCC discrimination are in line with our results [12,15,16]. The sensitivity reported in our study was the lowest, which can be explained by the rate of FN. Compared to RCM (62.5% sensitivity and 88.9% specificity) [24] and to OCT (87% sensitivity and 80% specificity) [18], LC-OCT showed much higher specificity in sBCC subtyping.

The main diagnostic pitfall encountered when discriminating BCC from BCC-imitators was the coexistence of sebaceous glands and BCC (the three FN in our study). Sebaceous glands can sometimes be mistaken for BCC lobules during a real-life examination, although they display different lobule morphology (granular-lobular vs. *millefeuille* pattern, respectively) [11,16,29]. Modern devices of LC-OCT, ensuring the complete coverage of the lesion via dermoscopic colocalization and providing diagnostic artificial intelligence assistance, will potentially avoid the occurrence of similar misdiagnoses [30].

False negatives in sBCC subtyping mostly occurred when trabecular superficial lobules were misinterpreted as branched lobules, an established predictor of iBCC [11], leading to an incorrect iBCC diagnosis on LC-OCT. Indeed, the 500 µm depth penetration of LC-OCT represents a limitation when differentiating these two subtypes. The enlargement of the aggregations of basaloid cells in superficial BCCs has been suggested to correlate with the clinical palpability of BCCs, making them difficult to diagnose by clinical and dermoscopic examinations as well [5]. We propose that when facing superficial branched structures, the end of the lobule, at its deepest part, should be carefully assessed. If the observer can delineate the deepest limits of the lobule, the diagnosis of trabecular sBCC rather than iBCC should be considered. If, on the contrary, the deepest limits are blurred or not clearly delineated, a deep trabecular sBCC or iBCC should then be considered, and the lesion should be excised. False positives in sBCC subtyping were also mainly due to the 500 µm penetration limit of LC-OCT, which hindered the detection of deeper nodular or infiltrative components. Nevertheless, the high PPV (92%) indicates that such errors are rare and that an LC-OCT diagnosis of sBCC is correct in the vast majority of cases.

Misclassifications in nBCC and iBCC subtyping may reflect discrepancies between LC-OCT and histopathology rather than true diagnostic errors. Histopathological subtyping is based on limited serial cutting of the specimen that may not capture the most representative/aggressive component visualised on LC-OCT. Conversely, LC-OCT allows for the in vivo assessment of the entire lesion, potentially detecting mixed patterns not evident in the histopathological examination. Moreover, differences in subtype definitions and terminology between LC-OCT and histopathology could potentially contribute to classification mismatches, particularly at the interface between nodular, micronodular and infiltrative growth patterns.

The impact of a misdiagnosis of BCC would depend on the correct diagnosis of the lesion: benign or malignant. The clinical impact of incorrectly subtyping BCC could mean not choosing the optimal treatment. It could mean that surgery would have been performed instead of a non-invasive treatment, in case of misdiagnosing a sBCC. Conversely, nBCC and iBCC, incorrectly subtyped as sBCC, could have undergone a probably unsuccessful non-invasive treatment instead of surgery.

Regarding the clinicians’ diagnostic confidence, LC-OCT increased by 2.7-fold the number of lesions diagnosed with high confidence compared to dermoscopy. This increase in confidence could translate clinically in physicians ruling in patients directly to treatment without doubt. Even though this analysis is subjective, in high-confidence lesions, diagnostic accuracy was higher, confirming the right judgment of the clinicians. In our study, ‘high diagnostic confidence’ was defined a priori as ≥90%, which is more than the ≥75% defined in the study by Gust et al. [14]; we intentionally adopted this more stringent cut-off to further challenge the added value of LC-OCT under conditions of very high observer certainty.

This study had the following limitations. The study protocol could not anticipate the challenge of the subtype reporting variability between pathologists and dermatologists. Another limitation was the low rate of BCC imitators: the observed prevalence of BCC (76%) was slightly higher than anticipated (70%), leading to 51 non-BCC included lesions instead of the expected 64; despite this, the power for the comparison of specificity between LC-OCT and dermoscopy remained robust. Moreover, subtype analyses were secondary, and the study was not powered enough for histologic subtype discrimination. Dermoscopic and LC-OCT evaluations in this study were performed by “expert observers” with at least three years of experience. Diagnostic performance may naturally differ for less experienced users; however, the future implementation of AI-assisted LC-OCT could enable novice users with AI support to achieve accuracy comparable to that of expert clinicians [30]. Moreover, LC-OCT assessments were not blinded to the clinical or dermoscopic context, which may have introduced observer bias; however, this design reflects real-world diagnostic practice and mirrors the routine use of non-invasive skin imaging. Furthermore, only equivocal lesions already planned for biopsy/excision were included, which may have introduced selection bias, as not all equivocal lesions may be systematically biopsied in real life; however, lesion selection/recruitment conformed with current guidelines stating that histopathological confirmation is mandatory in equivocal lesions [1].

## 5. Conclusions

To our knowledge, this is the first real-life prospective study reporting the diagnostic performance of LC-OCT for BCC diagnosis and subtyping performed at the patient’s bedside. We found an excellent diagnostic accuracy with a better specificity than dermoscopy, positioning this technique as a suitable tool to refine the diagnosis of equivocal lesions. It should be acknowledged that the penetration depth is still the main limitation of LC-OCT for BCC subtyping. In the diagnosis and subtype discrimination of BCC, LC-OCT could replace diagnostic biopsies, translating into a reduction in discomfort, waiting time and number of consultations for patients. This could further mean a potential reduction in costs to healthcare systems, but studies focusing on efficiency are needed to prove this hypothesis. Future extensions of the technology include the use of integrated and colocalised dermoscopy with the association of artificial intelligence to enable faster and more accurate diagnosis and subtype discrimination of BCC [30], as well as pre-surgical margin delineation.

## Figures and Tables

**Table 1:** Diagnostic performances to differentiate BCC from imitators.

			Histopathology			
			BCC	Non-BCC		Total
**Dermoscopy**	**BCC**		159	32		191
	**Non-BCC**		4	19		23
	**Total**		163	51		214
**LC-OCT**	**BCC**		160	5		165
	**Non-BCC**		3	46		49
	**Total**		163	51		214
**BCC vs.** **Non** **-BCC**	**Sensitivity**	**Specificity**	**Accuracy**	**PPV**	**NPV**	*p* *
**Dermoscopy**	98%	37%	83%	83%	83%	<0.001
**LC-OCT**	98%	90%	96%	97%	94%	

* MacNemar’s test (paired data). BCC—basal cell carcinoma; LC-OCT—line-field confocal optical coherence tomography; PPV—positive predictive value; NPV—negative predictive value.

**Table 2:** Diagnostic performances of dermoscopy and LC-OCT for BCC subtyping.

BCC Correctly Identified as Such on LC-OCT (*n* = 160)						
	Superficial BCC (*n* = 50)		Nodular BCC (*n* = 79)		Infiltrative BCC (*n* = 31)	
	Dermoscopy	LC-OCT	Dermoscopy	LC-OCT	Dermoscopy	LC-OCT
**Sensitivity**	62%	72%	81%	82%	6%	42%
**Specificity**	84%	97%	66%	75%	87%	82%
**Accuracy**	77%	89%	73%	79%	70%	74%
**PPV**	65%	92%	69%	76%	11%	36%
**NPV**	83%	88%	78%	81%	79%	85%
***p*** *	0.03		0.45		0.001	

* MacNemar’s test (paired data). BCC—basal cell carcinoma; LC-OCT—line-field confocal optical coherence tomography; PPV—positive predictive value; NPV—negative predictive value.

## Data Availability

The raw data supporting the conclusions of this article will be made available by the authors on request.

## Abbreviations

The following abbreviations are used in this manuscript:

BCC	Basal cell carcinoma
FN	False negative
FP	False positive
iBCC	Infiltrative basal cell carcinoma
LC-OCT	Line-field confocal optical coherence tomography
nBCC	Nodular basal cell carcinoma
NPV	Negative predictive value
OCT	Optical coherence tomography
PPV	Positive predictive value
RCM	Reflectance confocal microscopy
sBCC	Superficial basal cell carcinoma
